# Endothelial Semaphorin 7A Promotes Inflammation in Seawater Aspiration-Induced Acute Lung Injury

**DOI:** 10.3390/ijms151119650

**Published:** 2014-10-28

**Authors:** Minlong Zhang, Li Wang, Mingqing Dong, Zhichao Li, Faguang Jin

**Affiliations:** 1 Department of Respiration, Tangdu Hospital, Fourth Military Medical University, Xi’an 710038, China; E-Mails: 15991798305@163.com (M.Z.); wangli344@163.com (L.W.); 2 Department of Pathology and Pathophysiology, Fourth Military Medical University, Xi’an 710032, China; E-Mail: dongmqxh@fmmu.edu.cn

**Keywords:** Semaphorin 7A, acute lung injury, seawater

## Abstract

Inflammation is involved in the pathogenesis of seawater aspiration-induced acute lung injury (ALI). Although several studies have shown that Semaphorin 7A (SEMA7A) promotes inflammation, there are limited reports regarding immunological function of SEMA7A in seawater aspiration-induced ALI. Therefore, we investigated the role of SEMA7A during seawater aspiration-induced ALI. Male Sprague–Dawley rats were underwent seawater instillation. Then, lung samples were collected at an indicated time for analysis. In addition, rat pulmonary microvascular endothelial cells (RPMVECs) were cultured and then stimulated with 25% seawater for indicated time point. After these treatments, cells samples were collected for analysis. *In vivo*, seawater instillation induced lung histopathologic changes, pro-inflammation cytokines release and increased expression of SEMA7A. *In vitro*, seawater stimulation led to pro-inflammation cytokine release, cytoskeleton remodeling and increased monolayer permeability in pulmonary microvascular endothelial cells. In addition, knockdown of hypoxia-inducible factor (HIF)-1α inhibited the seawater induced increase expression of SEMA7A. Meanwhile, knockdown of SEMA7A by specific siRNA inhibited the seawater induced aberrant inflammation, endothelial cytoskeleton remodeling and endothelial permeability. These results suggest that SEMA7A is critical in the development of lung inflammation and pulmonary edema in seawater aspiration-induced ALI, and may be a therapeutic target for this disease.

## 1. Introduction

Acute lung injury (ALI) and its most severe form, acute respiration distress syndrome (ARDS), remains a leading cause for morbidity and mortality in critically ill patients [1]. Similar to stress situations such as sepsis, trauma and burns, seawater aspiration can also induce ALI/ARDS. Seawater aspiration-induced ALI/ARDS is characterized by severe dyspnea, serious hypoxemia and edema and these results are due to the effects of alveolar collapse, surfactant disruption and intrapulmonary shunting [2]. In the process of seawater aspiration-induced ALI, the inflammatory cells which have infiltrated into alveolar space contribute to the pulmonary inflammatory process through releasing several molecules, such as reactive oxygen species and pro-inflammatory cytokines [3,4]. It has been reported that the transmigration of inflammatory cells and formation of edema fluid are triggered by increased pulmonary microvascular endothelial permeability [5]. In addition, pro-inflammatory cytokines released by the stimulation of the immune system might be part of the hypoxia-enhanced expression of the nuclear factor kappa B (NF-κB) [6].

Semaphorins were originally identified as neuronal guidance proteins (NGPs) in the nervous system. The semaphorin family, characterized by a conserved *N*-terminal Sema domain (~500 amino acids), has been divided into eight subclasses based on sequence similarities and distinct structural features [7,8,9]. Semaphorin 7A (SEMA7A) is a membrane-associated GPI-linked semaphorin. In addition to inducing axon outgrowth, SEMA7A also holds the ability to promote the production of cytokines in inflammatory cells [10,11]. Moreover, SEMA7A can stimulate cytoskeletal reorganization in several types of cells such as melanocytes and monocytes, which can lead to spreading and migration of these cells [12,13]. In hypoxia, it has been observed that endothelial SEMA7A intensify inflammatory damage through promoting neutrophil trafficking and SEMA7A is regulated by the hypoxia-inducible factor (HIF)-1α [14]. However, there are limited reports regarding the expression and immunological function of SEMA7A during seawater aspiration-induced ALI.

In this study, we firstly investigated the lung injury and inflammation induced by seawater. In addition, we examined the influence of seawater on the regulation of SEMA7A and its role in the seawater aspiration-induced ALI both in lung tissues and rat pulmonary microvascular endothelial cells (RPMVECs). Our study identified that SEMA7A promoted the inflammatory damage and lung edema by increasing endothelial barrier permeability. Furthermore, this finding proved that SEMA7A is significantly induced by HIF-1α in seawater aspiration-induced ALI.

## 2. Results

### 2.1. Changes of Lung Histopathology and Pro-Inflammation Cytokines after Seawater Instillation

**Figure 1:**
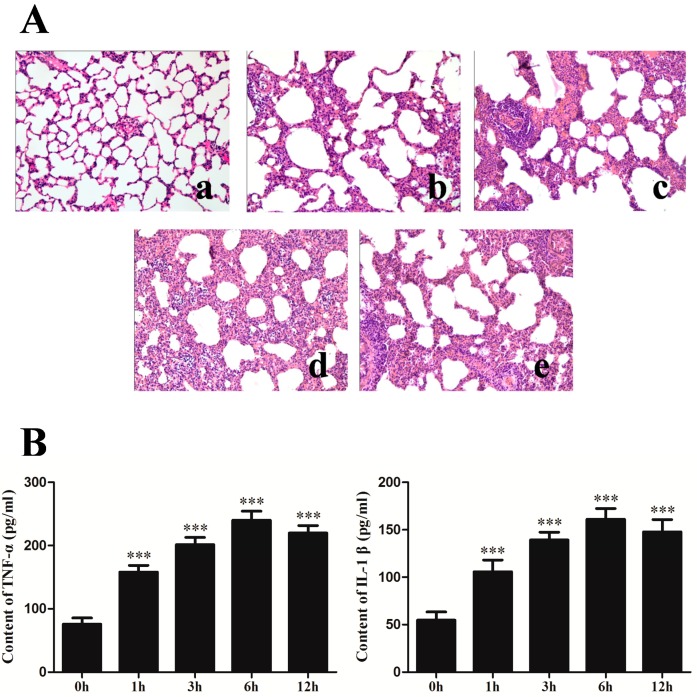
Changes of lung histopathology (**A**) and pro-inflammation cytokines expression (**B**) after seawater administration. (**a**–**e**) 0, 1, 3, 6 and 12 h seawater group. *n* = 8, *** *p* < 0.001 *versus* group **a**.

To investigate the level of lung injury after seawater stimulation, the hematoxylin–eosin (H–E) staining of lung tissues were performed (Figure 1A). Compared with control group, seawater instillation induced alveolar thickening, distortion and hemorrhage as well as pulmonary edema, infiltration of inflammatory cells in lung tissues and alveoli. Seawater induced lung injury reached its peak at 6 h. The pulmonary edema and infiltration of inflammatory cells were reduced in 12 h group compared with 6 h group.

To further evaluate the inflammation reaction after seawater administration, tumor necrosis factor (TNF)-α and interleukin (IL)-1β in the lung tissues were detected (Figure 1B). As shown in the figure, TNF-α and IL-1β significantly increased after seawater stimulation and reached their peak at 6 h (*p* < 0.001 compared with control group).

### 2.2. Rat Pulmonary Microvascular Endothelial Cell (RPMVEC) Characteristics

The primary cells were verified as endothelial cells via the capillary-like structure and typical cobble-stone appearance at confluence (Figure S1A). Sub-cultured cells were in accordance with endothelial cells in morphology. Furthermore, the sub-cultured cells displayed closely packed, homogeneous, short spindle or polygonal in shape (Figure S1B). Meanwhile, the isolated cells were confirmed as endothelial cells by positive expression of CD31 (Figure S1C).

### 2.3. Changes of Semaphorin 7A (SEMA7A) Expression in Seawater Aspiration-Induced Acute Lung Injury (ALI)

**Figure 2:**
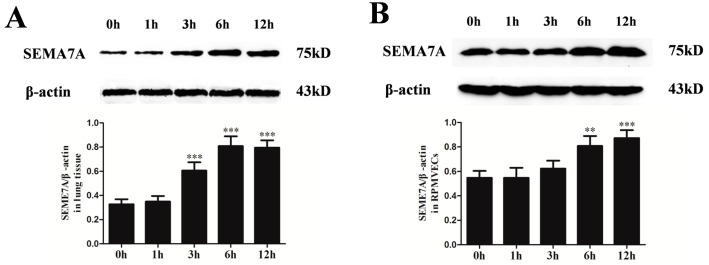
Changes of Semaphorin 7A (SEMA7A) expression in seawater aspiration-induced acute lung injury (ALI) in lung tissues (**A**) and rat pulmonary microvascular endothelial cells (RPMVECs) (**B**). Samples were obtained at 0, 1, 3, 6 and 12 h after seawater exposure. *n* = 8, ** *p* < 0.01, *** *p* < 0.001 *versus* 0 h group.

To study the changes of SEMA7A protein levels in response to seawater stimulation, we performed western blot both in rat lung tissues (Figure 2A) and RPMVECs (Figure 2B). As shown in the figure, SEMA7A protein expression significantly increased after seawater stimulation. The expression of SEMA7A reached its peak at 6 h and decreased at 12 h in lung tissues.

### 2.4. Specific siRNA against SEMA7A

To investigate the role of SEMA7A in the seawater aspiration-induced ALI, we silenced the expression of SEMA7A on RPMVECs by specific siRNA. As shown in the Figure S2, the specific siRNA significantly suppressed the transcription of SEMA7A (*p* < 0.001) and this effect was dose-dependent.

### 2.5. Endothelial SEMA7A Promotes Pro-Inflammation Cytokines Production in Seawater Aspiration-Induced ALI

**Figure 3:**
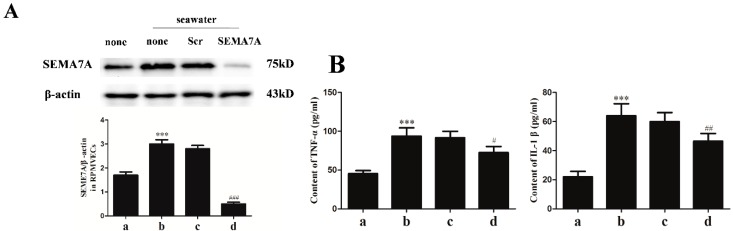
The siRNA suppressed the expression of SEMA7A elicited by seawater (**A**) and endothelial SEMA7A promotes pro-inflammation cytokines production in seawater aspiration-induced ALI (**B**). All data were obtained at 6 h after seawater administration. (a) normal group; (b) seawater group; (c) seawater + negative scrambled control (Scr siRNA) group; (d) seawater + SEMA7A siRNA group. *n* = 8, *** *p* < 0.001 *versus* group a, # *p* < 0.05, ## *p* < 0.01, ### *p* < 0.001 *versus* group b.

On the basis of increasing expression of SEMA7A elicited by seawater stimulation and given that seawater administration led to robust inflammatory responses, we explored the role of SEMA7A on the expression of pro-inflammation cytokines. We first silenced the expression of SEMA7A by transfecting RPMVECs with specific siRNA for SEMA7A. As shown in Figure 3A, the siRNA suppressed the expression of SEMA7A elicited by seawater (*p* < 0.001). Lack of the endothelial SEMA7A significantly reduced the expression of TNF-α and IL-1β (Figure 3B).

### 2.6. Endothelial SEMA7A Promotes Cytoskeletal Remodeling and Monolayer Permeability in RPMVECs Stimulated by Seawater

**Figure 4:**
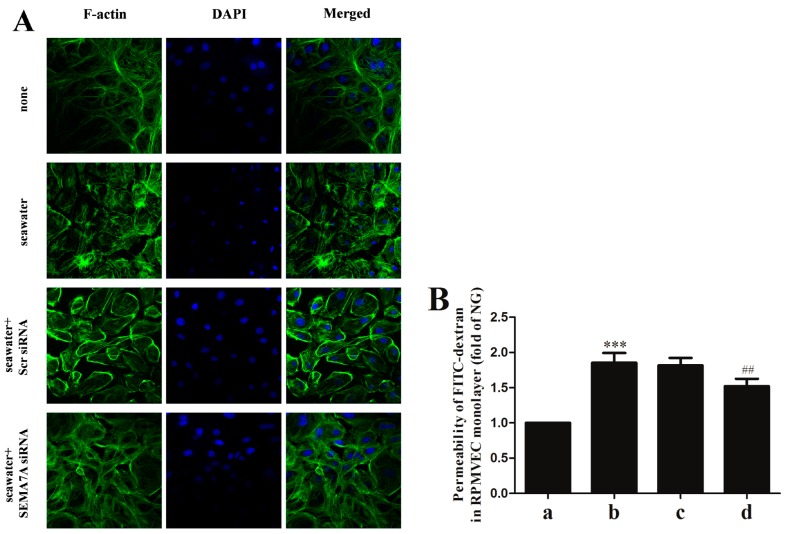
Endothelial SEMA7A promotes cytoskeletal remodeling and monolayer permeability in RPMVECs stimulated by seawater. All data were obtained at 6 h after seawater aspiration. (**A**) phenotype of cultured RPMVECs was examined by confocal microscopy following staining for F-actin (magnification 60×); (**B**) After RPMVEC monolayer formation, cells were treated with seawater. The permeability was shown as fold of normal group. (a) normal group; (b) seawater group; (c) seawater + negative scrambled control (Scr siRNA) group; (d) seawater + SEMA7A siRNA group. *n* = 8, *** *p* < 0.001 *versus* group a, ## *p* < 0.01 *versus* group b.

To assess the role of SEMA7A on cytoskeletal remodeling in RPMVECs, we stained F-actin on cells (Figure 4A). As shown in the figure, seawater exposure induced an evident motile phenotype characterized by increase of stress fibers and enlargement of gaps between cells. Lack of SEMA7A led to significant reduction of the actin-dependent cytoskeletal remodeling.

To investigate the role of SEMA7A on monolayer permeability of RPMVECs, we examined the fluorescein isothiocyanate (FITC)-dextran flux across the monolayer. As shown in Figure 4B, administration of seawater to cells resulted in a significant increase in FITC-dextran flux (*p* < 0.001 *versus* normal group). Lack of SEMA7A attenuated the increase of FITC-dextran flux (*p* < 0.01).

### 2.7. Hypoxia-Inducible Factor (HIF)-1α Mediated the Seawater-Stimulated Increase of SEMA7A in RPMVECs

**Figure 5:**
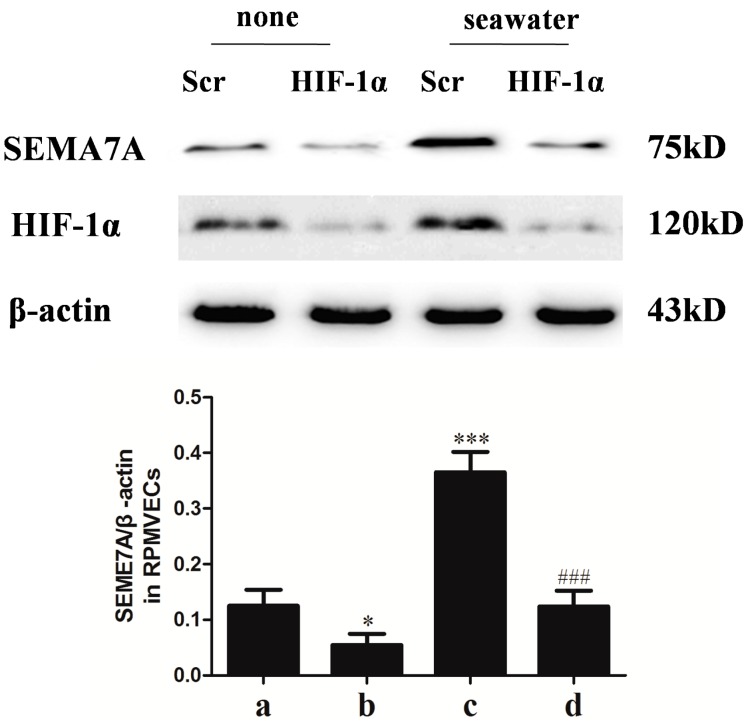
Hypoxia-inducible factor (HIF)-1α in endothelial SEMA7A induction in RPMVECs stimulated by seawater. All data were obtained at 6 h after seawater administration. (a) Negative scrambled control (Scr siRNA) group; (b) HIF-1α siRNA group; (c) seawater + negative scrambled control (Scr siRNA) group; (d) seawater + HIF-1α siRNA group. *n* = 8, * *p* < 0.05, *** *p* < 0.001 *versus* group a, ### *p* < 0.001 *versus* group c.

We further explored the role of HIF-1α in the seawater-stimulated increase of SEMA7A in RPMVECs via knockdown of HIF-1α with specific siRNA. As shown in the Figure 5, seawater administration significantly increased the expression of HIF-1α and SEMA7A. However, knockdown of HIF-1α with specific siRNA significantly inhibited the expression of SEMA7A in seawater stimulated RPMVECs.

## 3. Discussion

In this study, we explored the role of SEMA7A in seawater aspiration-induced ALI. The results showed that seawater stimulation led to lung histopathologic changes, lung edema, release of pro-inflammation cytokines and increase of endothelial barrier permeability. Furthermore, seawater stimulation also induced the expression of SEMA7A and this effect was through up-regulation of HIF-1α. Meanwhile, lack of the SEMA7A protected the endothelial barrier and inhibited the inflammatory reaction after seawater stimulation.

Seawater aspiration-induced ALI had two main characteristics: inflammation and pulmonary epithelial-endothelial barrier injury. Our previous studies have shown that seawater stimulation induced release of pro-inflammation cytokines and infiltration of inflammation cells. In the inflammation process, the NF-κB pathway was activated and HIF-1α expression was significantly induced [15,16]. Moreover, seawater also led to the pulmonary epithelial-endothelial barrier injury and the increase of lung tissue permeability as evidenced by bronchoalveolar lavage (BAL) fluid protein, lung wet/dry (W/D) ratio, the leak index of Evans blue and the recruitment of inflammatory cells [16,17,18].

SEMA7A was initially found to regulate neurite growth and axon track formation during embryonic development [19]. During recent years, numerous studies have shown that SEMA7A is also involved in immune responses. SEMA7A regulates inflammatory responses through modulation of T cell function, stimulation of macrophage recruitment and pro-inflammatory cytokine production, regulation of chemokine expression and dendritic cell migration [11,20,21]. During hypoxia-elicited lung inflammation, hypoxia induced expression of SEMA7A on endothelial cells and SEMA7A enhanced leukocytes trafficking through the endothelial layer [14]. Seawater aspiration stimulation also led to inflammation responses. In our present study, we explored the role of SEMA7A in seawater aspiration-induced ALI. We found that seawater induced lung injury and stimulated the expression of pro-inflammatory cytokines and inflammation responses were most obvious after 6 h of seawater exposure. Meanwhile, seawater administration increased the expression of SEMA7A with the extension of time both in lung tissue and RPMVECs and also up-regulated the expression of HIF-1α. However, suppression of SEMA7A inhibited the pro-inflammation cytokines production after seawater stimulation. Knockdown of HIF-1α inhibited the expression of SEMA7A. Considering this, we have demonstrated HIF-1α to be involved in the induction of SEMA7A during seawater stimulation. It has been observed that endothelial SEMA7A is regulated by HIF-1α during hypoxia [14] and our outcome is in accordance with this finding. Another critical issue of seawater aspiration-induced ALI is pulmonary edema. Several studies have confirmed that pulmonary edema and infiltration of inflammatory cells were resulted from endothelial barrier injury and endothelial permeability increase [22]. Our previous studies found that seawater stimulation can also induce endothelial barrier injury and increase barrier permeability via the detection of BAL fluid protein, W/D ratio and the leak index of Evans blue. Therefore, we hypothesized that SEMA7A promoted the inflammation and edema through increasing the endothelial permeability in seawater aspiration-induced ALI. We found that seawater stimulation induced the cytoskeletal remodeling and monolayer permeability in RPMVECs. Meanwhile, suppression of SEMA7A reduced the actin-dependent cytoskeletal remodeling and monolayer permeability. However, seawater aspiration-induced ALI is due to the effects of hyperosmolality and hypoxemia and it will be better to conduct the *in vitro* experiments under hypoxic conditions. The limitation of the *in vitro* experiments will be explored in the future work.

## 4. Experimental Section

### 4.1. Animals Preparation

Male Sprague–Dawley (SD) rats (5–7 weeks old) weighing 200 ± 20 g were obtained from the Animal Center of Fourth Military Medical University. The rats were kept in a temperature-controlled house with 12 h light-dark cycles and free access to standard laboratory diet and water *ad libitum*. All the animal experiments were approved by the Animal Care and Use Committee of the Fourth Military Medical University and in accordance with the Declaration of the National Institutes of Health Guide for Care and Use of Laboratory Animals (Publication No. 85–23, revised 1985).

The animals were randomly divided into five groups: normal control group, 1 h seawater group, 3 h seawater group, 6 h seawater group and 12 h seawater group. The seawater aspiration model was produced according to the method described in previous reports with some modifications. The rats were anesthetized with pentobarbital sodium (100 mg/kg, Sigma-Aldrich, St. Louis, MO, USA) intraperitoneally. The rats were maintained in the supine position during experiments with the head elevated 30°. A heparin-filled blunt-ended polyethylene catheter was inserted into the left carotid artery to monitor the mean arterial pressure and obtain blood samples. After exposure of the trachea, a 20 min stable baseline period was followed, and then a 1 cm syringe was gently inserted into the trachea approximately 1.5 cm above the carina. Next, seawater (4 mL/kg) was instilled at a steady speed within 4 min into both lungs. At the end of the experiment, the rats were exsanguinated by aortic transaction at 4 h after seawater instillation. The thorax was opened rapidly and lungs were processed in the manners described below. The rats in the normal control group were instilled with nothing.

### 4.2. Drug and Reagents

Seawater (osmolality 1300 mmol/L, pH 8.2, SW 1.05, NaCl 6.518 g/L, MgSO_4_ 3.305 g/L, MgCl_2_ 2.447 g/L, CaCl_2_ 1.141 g/L, KCl 0.725 g/L, NaHCO_3_ 0.202 g/L, NaBr 0.083 g/L) was prepared according to the major composition of the East China Sea provided by Chinese Ocean Bureau. Enzyme-linked immunosorbent assay (ELISA) kits for TNF-α and IL-1β were obtained from R&D Systems (Minneapolis, MN, USA). Antibody for SEMA7A was purchased from Abcam (Cambridge, UK). Anti-HIF-1α and anti-β-actin antibody were purchased from Santa Cruz Biotechnology Inc. (Santa Cruz, CA, USA). Other reagents were purchased from Sigma-Aldrich (St. Louis, MO, USA).

### 4.3. Lung Morphology

For lung histological studies, the rats were killed after seawater exposure. The same right lower lung lobes from every rat were preserved in 10% formalin for 24 h, and then embedded in paraffin wax, sliced and stained with hematoxylin–eosin (H–E). Microscopic evaluation was performed to characterize lung injury.

### 4.4. Measurement of Pro-Inflammation Cytokines

TNF-α and IL-1β in lung tissues or culture medium of RPMVECs were measured with ELISA kits according to the manufacturer’s protocol.

### 4.5. RPMVECs Culture and siRNA Transfection

Isolation and culture of primary RPMVECs were performed according to the previous methods with some modification [23,24]. Briefly, the fresh lung was aseptically removed from rats and washed. After the pleura and the outer edges of the lung lobe were cut off, the specimens of tissue (1.5 mm^3^) obtained from the lung surface were carefully plated into tissue culture dishes containing DMEM supplemented with 20% FBS, 100 U/mL of penicillin–streptomycin and 25 μg/mL of endothelial cell growth supplement (Upstate Biotechnology Inc., Lake Placid, NY, USA) at 37 °C in a humidified atmosphere containing 5% CO_2_ and 95% air. Sixty hours later, the residue lung tissues were removed. After that, the medium was changed every 2 days. When monolayer cells were achieved, the cells were passaged with a 0.25% solution of trypsin. Experimental data were obtained from cells between passage 2 and 3. Primary RPMVECs were identified according to their characteristic morphology such as staining with anti-CD31 antibody. Specific siRNA for rat SEMA7A and HIF-1α and the scramble control siRNA were purchased from Genechem (Shanghai, China). The siRNA was transiently transfected into RPMVECs with Lipofectamine 2000 (Invitrogen, Carlsbad, CA, USA) according to manufacturer’s instructions. The cells were stimulated with seawater (0.25 mL per 1 mL total volume) 48 h after transfection. The suppression efficiency was tested by RT-PCR and Western blot.

### 4.6. Determination of SEMA7A mRNA

Total RNA was extracted with TRIZOL reagent (Takara, Dalian, China) according to the manufacturer’s instruction. RNA concentration was tested by spectrometric analysis. SEMA7A and β-actin were examined by Real Time PCR following the manufacturer’s instructions (Takara Perfect Real Time). Amplification and detection were carried out by using Bio-Rad My iQ detection system (Edinburgh Biological Science and Technology Development Co.). Relative quantification of target cDNA was determined by arbitrarily setting the control value to 1 and changes in cDNA content of a sample were expressed as a multiple thereof. Genes and primers are listed as follows: The sequences of the rat *SEMA7A* primers were 5'-CGAGTGGCCCAGTTATGCAG-3' (forward) and 5'-AAACCAGCATGGCTTTCAGGA-3' (reverse). The sequences of the rat *β-actin* primers were 5'-ACGGTCAGGTCATCACTATCGG-3' (forward) and 5'-GCACTGTGTTGGCATAGAGGTC-3' (reverse).

### 4.7. Western Blot Analysis

In brief, the lysates extracted from the harvested lung tissues or cultured cells were removed by centrifugation at 12,000 rpm for 20 min at 4 °C. Protein concentration was determined by BCA protein assay kit. Protein was boiled in loading buffer, resolved in 10% SDS–polyacrylamide gels, electrotransferred to nitrocellulose membranes, and blocked with 5% non-fat milk in TBST. The membrane was incubated overnight with the indicated primary antibody β-actin (1:5000 dilution), and SEMA7A (1:1000 dilution). The secondary antibody (goat anti-rabbit IgG, 1:5000) was incubated and the relative content of target protein was detected by the enhanced chemiluminescent (ECL) detection system (Amersham Pharmacia Biotech, Arlington Heights, IL, USA) according to the manufacturer’s protocol.

### 4.8. Immunofluorescent Staining

For F-actin staining, cells were seeded on sterile glass slides and transfected with siRNA. Forty-eight hours after transfection, the cells were challenged with 25% seawater. Cells in serum-free growth medium were used as controls. Cells were then fixed with 4% paraformaldehyde (pH 7.4) for 10 min, permeabilized for 10 min with PBS containing 0.1% TritonX-100, and incubated with 2% bovine serum albumin (BSA) for 30 min. The F-actin was stained with fluorochrome-conjugated phalloidin (Alexa488-Phalloidin; Molecular Probes, Eugene, OR, USA) for 60 min at room temperature. Nuclei were stained with 4',6-diamidino-2-phenylindole (DAPI). Slides were mounted with ProLong Gold anti-fade reagent and read with confocal microscope.

### 4.9. Measurement of RPMVECs Monolayer Permeability

The permeability measurement was performed as previously described [25]. RPMVECs (2 × 10^5^ cells/well) were cultured on collagen coated Transwell insert to contrast an *in vitro* model of a cell monolayer. The insert were placed into 24-well plates containing 500 µL medium. After the cell monolayer formation and at the end of stimulation, 100 µL FITC-dextran (2000-kDa, Sigma) was added into the insert and incubated for 1 h. The insert was then removed and 100 µL medium collected from the bottom chamber. The fluorescent density of samples was analyzed by a fluorospectrophotometer (LS-50B, Bridgeville, PE, USA).

### 4.10. Statistical Analysis

All data are expressed as mean ± SD. Statistical comparisons were made using One-way Analysis of Variance (ANOVA) followed by Dunnett’s test. Differences were considered statistically significant at *p* < 0.05.

## 5. Conclusions

Our data demonstrated the role of SEMA7A in seawater aspiration-induced ALI. These findings showed that SEMA7A triggers the inflammatory responses by increasing the microvascular permeability and enhancing inflammatory cells’ recruitment during seawater aspiration-induced ALI. It may be considered as a potential therapeutic target for the treatment of seawater aspiration-induced ALI.

## Supplementary Materials

Supplementary figures can be found at http://www.mdpi.com/1422-0067/15/11/19650/s1.
